# Roles in brain stimulation, recording and interpreting neuroimaging data: a systematic survey of 26 countries on current practices in EEG, MRI, TCD, TMS and FUS

**DOI:** 10.3389/fmedt.2026.1917621

**Published:** 2026-09-04

**Authors:** Vugar Valizada, Leon Daði Sesseljuson, Raffaele Nardone, Aljoscha Thomschewski, Eugen Trinka, Yvonne Höller

**Affiliations:** 1 Faculty of Psychology, University of Akureyri, Akureyri, Iceland; 2 Department of Neurology, Hospital of Merano (SABES-ASDAA), Merano, Italy; 3 Department of Neurology, Neurocritical Care and Neurorehabilitation, Member of European Reference Network EpiCARE, Center for Cognitive Neuroscience, Christian Doppler University Hospital, Paracelsus Medical University, Salzburg, Austria

**Keywords:** EEG, focused ultrasound stimulation, MRI, transcranial Doppler, transcranial magnetic stimulation

## Abstract

**Introduction:**

Clear role definitions in performing and interpreting neurophysiological and neuroimaging procedures are essential for patient safety and diagnostic accuracy. There is currently no comprehensive overview of the international landscape of health professionals responsible for these tasks. In the present review, we aimed to summarize local practices for electroencephalography (EEG), brain magnetic resonance imaging (MRI), transcranial magnetic stimulation (TMS), transcranial Doppler (TCD), and focused ultrasound stimulation (FUS).

**Methods:**

We systematically searched scientific literature, national health organizations websites, and contacted local health authorities from 26 countries.

**Results:**

EEG and brain MRI are typically performed by technicians and interpreted by physicians (neurologists or radiologists). TCD is performed and interpreted predominantly by physicians, since it requires real-time evaluation. Single-pulse TMS is either performed by physicians or requires the presence of physicians when the procedure is performed by a technician. The interpretation is done by physicians. Despite existing international recommendations, there is a significant absence of local guidelines for responsibilities in performing and interpreting TMS and FUS. In countries with information available, therapeutic use of rTMS, and FUS is performed by physicians (neurologists, psychiatrists, or surgeons).

**Discussion:**

The development of standardized national protocols is needed to ensure patients' safety and diagnostic accuracy in clinical settings for TMS and FUS.

## 1 Introduction

Electroencephalography (EEG), magnetic resonance imaging (MRI), transcranial Doppler ultrasound (TCD), transcranial magnetic stimulation (TMS), and focused ultrasound (FUS) are essential tools in clinical and applied neurosciences. The costs associated with these modalities are not only determined by the actual technical equipment and infrastructure, but also by the specialized staff required to operate the technology and interpret the results. Variations in professional responsibilities therefore have direct implications for healthcare expenditures and accessibility of diagnostic services.

EEG is an essential tool for measuring the brain's electrical activity. Even though more advanced imaging techniques were developed, EEG defends its role as an indispensable but, yet inexpensive and highly indicative method for diagnosing epilepsies and specifically detecting seizures or interictal epileptiform activity (1). Additionally, the EEG is also used for evaluating encephalopathies (2), coma (3, 4) and non-convulsive status epilepticus (5–7), it serves as a biomarker for dementia (8), depression (9), and sleep disorders (10). As stated by International Federation of Clinical Neurophysiology (IFCN) and International League Against Epilepsy working group, the procedure of obtaining EEG data itself is usually performed by an EEG technician after which the recordings are viewed and evaluated by a board-certified neurologist with subspecialty in EEG and often also expertise in epilepsy (11, 12). Recording and saving the obtained data implies that EEG records can be reviewed later (13). An alternative scenario is the situation when a patient arrives with suspected status epilepticus or other critical neurological conditions, and the EEG is used for online monitoring of the effectivity of medication (14).

Transcranial Doppler (TCD) is a non-invasive, painless test that measures the speed and direction of the blood flow in brain arteries using ultrasound through acoustic windows, thin spots in the skull. Since its introduction in 1982, TCD has been widely applied in outpatient and inpatient settings (15). It is used for the diagnosis of a variety of cerebrovascular disorders such as ischemic stroke (16), vasospasm after subarachnoid hemorrhage (17), and sickle cell disease (18). According to American Institute of Ultrasound in Medicine guidelines TCD is performed by a sonographer or a physician trained in this technique (19, 20). Although the procedure itself is inexpensive and the device is portable, the result is heavily dependent on the sonographer responsible for performing it (21, 22).

MRI of the brain is a well-established, non-invasive procedure for evaluating neurological conditions (23). It is crucial for diagnosing disorders such as multiple sclerosis (24), stroke (25), neurodegenerative diseases (26), and encephalopathies (27). Brain MRI includes several sequences, such as T1-weighted, T2-weighted, FLAIR, diffusion-weighted imaging (DWI), apparent diffusion coefficient (ADC) mapping, gradient echo and susceptibility weighted imaging (SWI), all with their specific indications for the diagnosis and prognosis of neurological conditions (28, 29). More specifically, the choice of sequences depends on the clinical setting. In an acute context (for example, suspected stroke), faster protocols focusing on DWI/ADC and SWI are often used (30, 31). In planned or elective MRI studies such as multiple sclerosis, encephalopathy, or neurodegenerative disorders, more comprehensive sequences are incorporated (32). These extended protocols increase the acquisition time, which in some cases may take 20 min to one hour (33). In addition to duration, the drawbacks of brain MRI include cost and limited availability of MRI scanners in many hospitals (34). According to American College of Radiology the procedure is performed by technologist and interpreted by licensed radiologist (35).

Transcranial magnetic stimulation (TMS) is a non-invasive procedure that uses magnetic fields to examine the integrity of neural circuitry in neurologic and psychiatric diseases (36). There are two main types: single-pulse TMS and repetitive TMS (rTMS) (37).

Single-pulse TMS is primarily used for diagnostic purposes. It can elicit motor evoked potentials (MEPs), which are recorded from target muscles using surface electromyography (EMG) (38). The measured transmission delays and patterns can be used to assess corticospinal tract integrity and cortical excitability (39).

Repetitive TMS (rTMS) is mainly used as a therapeutic intervention (40), where the frequency of stimulation determines its effect. Low frequency (<1 Hz) rTMS inhibits cortical excitability, but high frequency (>5 Hz) rTMS enhances cortical excitability (41–43). rTMS has been shown to modulate neuronal activity and is used in the management of several conditions, including major depressive disorder (44), post-stroke motor rehabilitation (45), neuropathic pain (46, 47), or obsessive-compulsive disorder (OCD) (48). Drawbacks of rTMS include that it takes many sessions, such that it is relatively time-consuming (often requiring 20–30 daily sessions) until clinical effects are measurable (49). Additionally, rTMS as a therapy has limited availability in many countries due to costs and the need for specialized equipment and trained personnel (50, 51).

The IFCN guidelines describe three main categories of professionals involved in TMS operations (42, 52). According to this guideline, technicians are responsible for carrying out TMS sessions with patients or research participants, monitoring their safety and comfort, and administering certain standardized assessments (such as depression rating scales). Physicians determine whether TMS is appropriate for a patient, select and prescribe the most suitable protocol, and oversee the work of technicians. Scientists are typically the principal investigator or a senior co-investigator in research projects or clinical trials involving TMS. They design and implement the study protocol, either conducting the sessions themselves or supervising technicians.

Another promising and highly interesting technology of brain stimulation is FUS. It is praised for its clinical potential in neurological settings, oncological disorders, and for pain management (53). FUS as an interventional procedure has been approved by the FDA for treatment of uterine fibroids and bone metastases (54), as well as for thalamotomy in essential tremor and tremor-dominant Parkinson's disease (55). Transcranial FUS is divided into the subfields of high- (>200 W/cm^2^), medium- (100–200 W/cm^2^), and low-intensity (<100 W/cm^2^) FUS (56). High-intensity FUS creates permanent lesions through coagulation/thermal ablation and is therefore considered a surgical technique. Medium-intensity FUS allows breaking through the blood-brain barrier for drug delivery e.g., as demonstrated in neurodegenerative diseases (57). Low-intensity FUS exhibits non-destructive mechanical pressure on cellular membranes and ion channels, and thereby modulates brain activity, e.g., as demonstrated through neuromodulation effect in patients with drug resistant epilepsy in Phase 1 pilot trials (58). Drawbacks of FUS include the still high costs of the procedure and the circumstance that many potential clinical applications are still in the investigational or clinical trial phase (59).

Established methods of EEG, MRI, and TMS-MEP are operated by designated staff in healthcare institutions, while experimental methods such as rTMS and FUS are often provided only in a research setting. FUS is a very young method and, while gaining momentum, the roles and responsibilities for delivering FUS remain unclear. Understanding which medical professionals are most likely to perform and interpret the procedure in the future is of particular importance for the planning of studies and healthcare budgets, and for establishing routines and research collaborations. It is likely that similar professional responsibilities as for EEG, MRI, and TMS will be established for FUS in the future. Therefore, recommendations for studies on FUS should be based on the best clinical practice and/or guidelines for existing methods. However, while responsibilities are regulated in international guidelines, there is limited evidence providing a comprehensive overview of national practices that stakeholders, such as companies, could use to determine the target users for a future medical device.

Therefore, the present systematic survey addresses the research question which medical professionals are responsible for performing and interpreting methods that are standard in clinical practice (EEG, MRI, TCD, TMS-MEP) and less established (rTMS) procedures in healthcare systems of different countries. We included well-established and regulated technologies (EEG, MRI) as well as less-regulated ones (TMS, FUS) to illustrate differences in existing practices and how these differences can further help identify end users for novel technologies such as FUS. Our intention is not to directly compare these modalities on the same level, but rather to examine current practices in neurotechnological modalities to identify how responsibilities and user roles are defined across technologies with different levels of establishment. Obtaining reliable data on existing technologies and current practices in public healthcare systems will allow to give recommendations for the development of guidelines and best practice for futuristic technologies, especially FUS, for clinical purposes. In detail, the present review addressed the following research questions:

- Which health professionals are responsible for recording health records via EEG, MRI, TCD, and TMS?
- Which health professionals are responsible for evaluating recorded data via EEG, MRI, TCD, and TMS for diagnostic purposes?
- Which health professionals apply rTMS for therapeutic purposes?
- Which health professionals are the most likely target users of FUS for therapeutic interventions?

In this article, we are using the following terms: “performing/recording” and “interpretation”. By “performing/recording” we refer to data acquisition in one or more modalities. By “interpreting” we refer to the process of reviewing acquired imaging or neurophysiological data, describing the findings and drawing conclusions which will lead to diagnostic or therapeutic decisions.

## 2 Methods

Case studies for this survey were defined as countries across five continents: North America (Canada, USA), South America (Argentina, Brazil), Europe (Austria, Denmark, France, Iceland, Italy, Germany, Netherlands, Germany, Poland, Russia, Serbia, Spain, UK), Asia (China, India, Japan, Turkey, UAE), Africa (Algeria, Egypt, Nigeria, Republic of South Africa), and Australia. These countries were selected based on an initial screening on availability of information and the availability of the technologies of interest. To this end, we also consulted the Global Atlas of Medical Devices by the WHO (60), which provides information on availability of MRI, among others.

We searched scientific databases (PubMed, Cochrane Central) as well as the general web to retrieve information.

### 2.1 Level of trust

Sources of information were categorized into two groups based on their reliability:

*High trust*: National health organizations, hospital websites, direct correspondence with local health authorities, peer-reviewed articles.
*Low trust*: General websites, online forums, and unofficial reports.

We sought to establish high trust information for each country to enhance data accuracy.

### 2.2 Pubmed and Cochrane Central

The systematic search in PubMed was conducted by a physician specialized in Neurology.

#### 2.2.1 EEG

For determining staff involved in recording and evaluating the EEG we performed a search on Pubmed on 20th October 2025, with the terms “(((EEG recording) OR (EEG interpreting) or (EEG procedure)) AND (((technician) OR (nurse) OR (neurologist) OR (neurophysiologist)))) AND (guideline)”. This strategy of narrowing down the focus on recording and interpretation of EEG resulted in a list of 49 articles. After screening titles and abstracts, 43 records were excluded, and 6 articles were assessed as eligible to provide information for the present purpose. On 21st July 2026 we conducted search in Cochrane Central with following terms (“EEG recording” OR “EEG interpreting” OR “EEG interpretation” OR “EEG procedure” OR electroencephalography) AND (technician* OR nurse* OR neurologist* OR neurophysiologist*) AND (guideline* OR recommendation* OR consensus OR standard* OR protocol*). Out of 75 results 10 were eligible for our study.

#### 2.2.2 MRI

The search terms “(((brain MRI performing) OR (brain MRI interpreting) OR (brain MRI procedure)) AND ((technician) OR (technologist) OR (radiologist) OR (neuroradiologist))) AND (guideline)” were used for search on PubMed on 22nd October 2025. This search produced 57 results, among which 36 articles were excluded. The remaining 21 articles were evaluated as pertinent for our study. Following search terms (“brain MRI” OR “brain magnetic resonance imaging”)AND (technician* OR technologist* OR radiologist* OR neuroradiologist*) AND (guideline* OR recommendation* OR consensus OR standard* OR protocol*) were used on 23rd July 2026 for the Cochrane Central search. The search returned 24 hits; 1 of the results was pertinent to our study.

#### 2.2.3 TCD

The following search terms were used “(transcranial doppler ultrasound) AND (guideline)” for a broad search on PubMed on 23rd October 2025 which delivered 89 hits and “(((transcranial doppler ultrasound performing) OR (transcranial doppler ultrasound interpreting) or (transcranial doppler ultrasound procedure)) AND (((technician) OR (technologist) OR (radiologist) OR (sonographer) OR (physician)))) AND (guideline)” for the second search on 24th October 2025 which delivered 16 hits. 16 of the results were overlapping. 76 records were excluded based on title and abstract. 13 were assessed as eligible and studied for the present purpose. For the Cochrane Central search, we used the terms (“transcranial Doppler” OR TCD OR “transcranial Doppler ultrasound” OR “transcranial ultrasonography” OR “transcranial ultrasound”) AND (technician* OR technologist* OR sonographer* OR radiologist* OR physician*) AND (guideline* OR recommendation* OR consensus OR standard* OR protocol*) on 23rd July 2026. The search yielded 22 results, none of which were eligible for our study.

#### 2.2.4 TMS

Firstly “(transcranial magnetic stimulation) AND (guideline))” search terms were used on PubMed on 26th October 2025, which delivered 125 results and “((transcranial magnetic stimulation) OR (transcranial magnetic stimulation procedure) OR (rTMS) OR (MEP-TMS) AND (((technician) OR (technologist) OR (physician) OR (neurologist) OR (psychiatrist)))) AND (guideline)” was used for the second search, also on 26th October 2025, which delivered 18 results. 18 records were overlapping, 107 were excluded based on title and abstract and 18 were assessed as eligible for the study. On 24th July 2026 we searched the Cochrane Central database with following terms (“transcranial magnetic stimulation” OR TMS) AND (technician* OR technologist* OR physician*)AND (guideline* OR recommendation* OR consensus OR standard* OR protocol*). Out of 86 results, 13 were deemed eligible for our study.

#### 2.2.5 FUS

The first search using “(focused ultrasound) AND (guideline) AND (application)” on PubMed on 27th October 2025 yielded 112 results. A second search, also on 27th October 2025, with following terms “(focused ultrasound) AND (guideline) AND (application) AND ((surgeon) OR (physician) OR (clinician) OR (technician) OR (researcher))”, produced 73 results, all overlapping with the initial set. After screening, 112 records were excluded, and no articles were identified as eligible for the study's purposes. For the Cochrane Central database we used following terms (“focused ultrasound” OR FUS) AND (surgeon* OR physician* OR clinician* OR technician* OR researcher*)AND (guideline* OR recommendation* OR consensus OR standard* OR protocol*) on 25th July 2026. Of the 42 results, no articles were deemed pertinent to our study.

### 2.3 Google search

Since information on country specific clinical practice rules is not necessarily available in the form of scientific publications, we additionally performed a google search to regional national rules and regulations. Initial data were gathered by two undergraduate students in Psychology (03.2023–05.2023) and subsequently reviewed and validated by a physician specialized in Neurology (01.08.2025–30.09.2025).

For each country, information was sought through the following search terms in different languages:

“EEG” “brain MRI” “TCD” “TMS” “procedure” “guideline” “interpret” “perform” “overview” “challenges” “insurance” “availability”.

Data collection covered sources in English, German, Italian, Icelandic, Russian, and Turkish, which were directly interpreted by the research team. For other languages, Google translate was used.

### 2.4 Efforts to retrieve missing information

If the search strategies did not yield high-trust information, we sent emails to national health authorities, neurology societies, and radiology societies. After sending each email, we waited 8 weeks for a reply. Direct correspondence was particularly important for information on transcranial Doppler ultrasound and transcranial magnetic stimulation, as publicly available data was limited. Please see the Supplementary Tables for further details on the sources.

## 3 Results

### 3.1 Level of trust

Level of trust information is given in Table 1. Links to sources are provided in the Supplementary Tables.

**Table 1:** Trustworthiness of the source.

Country	EEG	MRI	TCD
USA	A	A	A
Canada	A	A	A
Brazil	A	B	B
Argentina	B	Missing	Missing
UK	A	A	A
Germany	A	A	A
France	A	B	A
Austria	A	A	A
Netherlands	A	A	A
Denmark	A	A	A
Iceland	Missing	A	Missing
Italy	A	B	B
Spain	A	A	A
Poland	B	B	B
Serbia	B	Missing	Missing
Turkey	A	A	B
Russia	A	A	A
India	A	A	A
China	A	Missing	Missing
Australia	A	A	A
Japan	A	Missing	Missing
UAE	A	A	Missing
Algeria	Missing	Missing	Missing
Egypt	Missing	Missing	Missing
Nigeria	Missing	B	Missing
RSA	A	A	A

A: high trust, B: low trust, Missing: no information was available.

For EEG we could obtain high trust (A) information from 19 countries and for MRI from 15 countries. These countries included predominantly countries from North America, Western Europe, and Australia.

For TCD, the available information was more limited. In the case of 13 countries (Australia, Austria, Canada, Denmark, France, Germany, India, Netherlands, RSA, Russia, Spain, UK, USA) we could obtain high trust (A) information. Specifically, for Australia, Austria, Denmark, Germany, RSA, Russia and Spain the information was obtained through correspondence with health authorities.

For TMS, even less data was available. It was only possible to gain high trust (A) information for 3 countries (Australia, UK, USA) and low trust (B) information for 4 countries (Denmark, Germany, Netherlands, Japan).

No information was found for FUS.

### 3.2 Responsibilities across countries

Information on recording and interpreting responsibilities is given in Tables 2, 3, respectively.

**Table 2:** Responsibilities for recording data across countries.

Country	EEG	MRI	TCD
USA	Technician	Technician	Technician/physician
Canada	Technician	Technician	Technician/physician
Brazil	Technician	Technician	Technician/physician
Argentina	Technician	Missing	Missing
UK	Technician	Technician	Technician/physician
Germany	Technician	Technician	Physician
France	Technician	Technician	Physician
Austria	Technician	Technician	Physician
Netherlands	Technician	Technician	Physician
Denmark	Technician	Technician	Technician
Iceland	Missing	Technician	Missing
Italy	Technician	Technician	Technician/physician
Spain	Physician	Technician	Physician
Poland	Technician	Technician	Missing
Serbia	Technician	Missing	Missing
Turkey	Technician	Technician	Physician
Russia	Technician	Technician	Physician
India	Physician	Technician	Technician
China	Technician	Missing	Missing
Japan	Technician	Missing	Missing
Australia	Technician	Technician	Technician/physician
UAE	Technician	Technician	Missing
Algeria	Missing	Missing	Missing
Egypt	Missing	Missing	Missing
Nigeria	Missing	Missing	Missing
RSA	Technician	Technician	Physician

**Table 3:** Responsibilities for interpreting data across countries.

Country	EEG	MRI	TCD
USA	Physician	Physician	Physician
Canada	Physician	Physician	Physician
Brazil	Physician	Physician	Physician
Argentina	Physician	Missing	Physician
UK	Physician	Physician	Physician
Germany	Physician	Physician	Physician
France	Physician	Physician	Physician
Austria	Physician	Physician	Physician
Netherlands	Physician	Physician	Physician
Denmark	Physician	Physician	Physician
Iceland	Missing	Physician	Missing
Italy	Physician	Physician	Physician
Spain	Physician	Physician	Physician
Poland	Physician	Physician	Physician
Serbia	Physician	Missing	Missing
Turkey	Physician	Physician	Physician
Russia	Physician	Physician	Physician
India	Physician	Physician	Physician
China	Physician	Missing	Missing
Japan	Physician	Missing	Missing
Australia	Physician	Physician	Physician
UAE	Physician	Physician	Missing
Algeria	Missing	Missing	Missing
Egypt	Missing	Missing	Missing
Nigeria	Missing	Physician	Missing
RSA	Physician	Physician	Physician

For EEG and brain MRI, a consistent pattern was observed. The procedure of data collection is performed by non-medical doctors such as technologists, while the interpretation is carried out by neurologists and radiologists, respectively.

For TCD the practices were profoundly different between countries. In some countries (USA, Canada, UK and India) the responsibilities were split: technicians were responsible for performing TCD and physicians for interpreting results, but in other countries (Austria, Denmark, France, Germany, Russia and Spain) physicians were responsible both for performing and interpreting TCD.TMS was approved by the U.S. Food and Drug Administration (FDA) in 2008 and is currently covered by insurance in several countries, including the Australia, Denmark, Germany, Japan, Netherlands, UK and the United States. According to existing guidelines, TMS should be performed by a technician under the supervision of a physician (42, 52).

When it comes to FUS as a therapeutic tool, there is no division in responsibilities for performing/interpreting. Since many potential clinical applications are in the investigational or clinical trial phase, we could not find clear guidelines for operating the technology.

## 4 Discussion

With this research, we aimed to determine the distribution of roles in recording and interpreting data obtained with specific neurological diagnostic technologies and in performing interventional brain stimulation procedures. Our intention was not to compare international and national guidelines, but rather to investigate who is responsible for performing and interpreting each modality in a selection of countries. We contacted health authorities and national neurological and radiological societies, while simultaneously searching the PubMed database and the general web. Following an eight-week waiting period for responses, the obtained information was categorized into two groups based on its level of trustworthiness, as described above. For each country, the most trustworthy source among the gathered information was selected for further analysis. According to our survey, despite the existence of international guidelines, there is a widespread lack of local and national protocols for the usage of some established methods.

### 4.1 Responsibilities of recording EEG, MRI, TCD, and TMS

In the case of EEG and brain MRI, technicians record/perform the procedure. TCD, on the other hand, is predominantly performed by physicians, as real-time evaluation of blood vessels is essential. Single-pulse TMS as used for diagnostic purposes, according to existing guidelines, must be performed either by a technician in the presence of a physician or by physicians themselves (52). We could not find local guidelines for TMS, which is most likely explained by its currently limited use (50, 51).

### 4.2 Responsibilities for evaluating EEG, MRI, TCD, and TMS

In all countries included in this survey, interpretation of EEG and brain MRI results is done by physicians—neurologists or clinical neurophysiologists for the former, and radiologists or neuroradiologists for the latter. TCD is also interpreted by physicians. However, since it is used both for neurological conditions and for sickle cell anemia, it may be interpreted by neurologists, pediatricians, intensivists, or other physicians with expertise in sonography. Single-pulse TMS is also recommended to be interpreted by a physician according to the guidelines, although local protocols that would demonstrate compliance with these guidelines are lacking (52).

### 4.3 Which health professionals apply rTMS for therapeutic purposes?

According to our data, rTMS as a therapeutic procedure is performed by physicians. rTMS is applied in the treatment of psychiatric and neurological disorders (40) which implies that either a psychiatrist or a neurologist is responsible for conducting the procedure. For example, rTMS is used to treat major depression (44) and OCD (48), to alleviate neuropathic pain (46, 47), and to improve post stroke rehabilitation (45). However, the disadvantage of a physician applying the treatment is clearly that clinics already lack manpower in general, and particularly with limited availability of physicians, nurses, and technicians. On an expert level. Hence, the practice that rTMS is only applied by physicians is also limiting the availability of the method to a wider range of patients, because of the high costs of paying physicians to employ it but also because of the limited time resources available that physicians can devote to applying rTMS. For example, to treat depression a typical therapeutic plan includes 20–40 treatment sessions over 4–8 weeks, each lasting about 45 min (49). To allow more patients to benefit from the positive effects of rTMS it is of utmost importance that more practical solutions are found, e.g., the establishment of clinical curricula for medical professionals that provides a staff license allowing them to apply the procedure without the need to drain existing scarce personnel resources but holding up highest standards of patient safety.

### 4.4 Which health professionals are the most likely target users of FUS for therapeutic interventions?

There are very few FDA-approved indications for FUS. Specifically, all of them regarding high-intensity FUS and are performed by physicians, mostly surgeons (54, 55). For example, clinical evidence provides the grounds for the FDA-approved use of FUS for surgical treatment of uterine fibroids and bone metastases (54). While many potential clinical applications of FUS are still in the clinical trial phase (59), there is a need for standardized guidelines for operation of this device, specifically for medium- and low-intensity FUS. The license to apply high-intensity FUS to brain tissue are and should be reserved to neurosurgeons as the procedure causes permanent changes in the brain. In contrast, the responsibilities for medium- and low-intensity FUS need to be determined after sufficient evidence for the safety and therapeutic value of the procedure is generated. At the present status, it is too early to call for medical health professionals to be specialized in FUS and the limitation to medical doctors being the sole experts allowed to apply the method at any intensity is important. However, for future scenarios there should be discussions on dedicated educational pathways for low-intensity FUS, which do not cause permanent changes but only temporary neuromodulation. Like for rTMS, that would open novel treatment horizons for difficult to treat conditions such as drug-resistant epilepsy (58) and substance use disorder (61) which were shown to benefit from low-intensity FUS.

### 4.5 Limitations

This study has several limitations. The most striking limitation is that while being based on a thorough systematic search of scientific databases and targeted search for national guidelines via general web searches, a specific polling and evaluation of national laws as source documents was not included. This approach would have required the inclusion of local law experts who are proficient in their respective languages and familiar with the local organization of laws and regulations in the field.

Secondly, only the PubMed databases and Google search engine were used to retrieve information. While these are commonly used search engines that give a broad selection of scientific and non-scientific records, other databases could have led to retrieval of more resources.

Thirdly, technologies considered in this analysis differ substantially in their historical development and usage. While EEG and MRI have been established for decades, FUS is comparatively recent. As a result, responsibilities and target users may not be equally well defined, which should be taken into consideration when interpreting the results of this study. However, this is per-se part of the scope of the study to determine the level of regulation and roles taken for the respective technology at different stages of establishment.

Fourthly, we aimed to provide an overview of existing practices, focusing on the roles involved in performing and interpreting different modalities, rather than a comparative analysis of these recommendations. A more detailed analysis of these underlying factors was beyond the scope of the present work but represents an important area for future research.

Fifthly, we acknowledge that intra-national differences in guidelines may exist, which could affect and, to some extent, limit reproducibility. In particular, variations may arise due to the general shortage of healthcare personnel in hospitals, potentially leading to differences in guideline implementation within the same country. For instance, according to World Health Organization data, there are fewer than 0.1 neurologists per 100,000 population in Africa and 0.1 per 100,000 in South-East Asia, compared to 6.6 per 100,000 in Europe (62). This marked disparity, further combined by shortages of trained technicians, may partly explain the possible differences in intra-national guidelines (63)*.* Another contributing factor may be the elevated costs associated with educating and training technicians (64).

A sixth limitation applies particularly to the case of FUS. Because of the low availability of data, we focused on high-intensity FUS, since there are no FDA-approved clinical applications for low-intensity FUS.

Another limitation can be construed from the limited list of technologies included in this review. For example, other innovative technologies such as transcutaneous Vagus Nerve Stimulation (tVNS) or transcranial direct current stimulation (tDCS) were not included although they are less expensive as compared to TMS and FUS. However, the technologies included in this review were selected to represent complementary approaches for structural, functional, and cerebrovascular assessment (MRI, EEG, and TCD), together with focal neuromodulation techniques (TMS and FUS). Although other non-invasive neuromodulation methods, such as tDCS and tVNS, are clinically relevant and more affordable—particularly in low- and middle-income settings—they were beyond the scope of this review because our focus was on modalities with established roles in multimodal neuroimaging integration and/or high spatial precision for targeted brain modulation. Another consideration underlying our selection is the intended clinical context of the technologies. tDCS and tVNS are portable neuromodulation modalities that, with appropriate patient selection, training, and clinical supervision, can be administered in home-based settings. In contrast, TMS and FUS require specialized equipment, trained personnel, and treatment within dedicated clinical facilities to ensure accurate targeting, treatment delivery, and patient safety.

Finally, even though information in languages English, German, Italian, Icelandic, Russian, and Turkish was directly interpreted by the multinational research team, for sources in other languages Google translate was used, which might have yielded unreliable translations in some cases. Future research should expand the list of countries and include multiple databases.

## 5 Conclusion

With this study we aimed to collect and compare local practices and guidelines for role division in interpreting and operating different modalities. Although it was possible to obtain trustworthy information on responsibilities for EEG and brain MRI, we could not find reliable information for TCD, TMS, and FUS for all countries included in this survey. We claim that for these modalities there is a need to develop and distribute international and national guidelines that are accessible, because such information will help not only local health authorities, but also manufacturers who are looking for stakeholders and target users of their systems. Identifying the future user group of medical professionals applying these technologies will help for smooth transition of interventions leveraging TMS and FUS from research to clinical settings. Additionally, to pave the way to clinical practice for therapeutic interventions with rTMS and low- to medium-intensity FUS, clinical curricula need to be established to educate professional staff beyond those holding a medical degree in safe administration, allowing for a more cost-effective therapeutic offer and increase availability of these promising procedures.

## Data Availability

The original contributions presented in the study are included in the article/Supplementary Material, further inquiries can be directed to the corresponding author.
