# Supplementary material for: Roles in brain stimulation, recording and interpreting neuroimaging data: a systematic survey of 26 countries on current practices in EEG, MRI, TCD, TMS and FUS
Source: Front Med Technol. 2026 Sep 4;8:1917621. doi: 10.3389/fmedt.2026.1917621 (PMC13585712; doi:10.3389/fmedt.2026.1917621)
Supplement: Supplementary file 1 [file Datasheet1.pdf]

Roles in brain stimulation, recording and interpreting neuroimaging data: A systematic survey of 26 countries on current practices in EEG, MRI, TCD, TMS and FUS

Vugar Valizada[1*], Leon Daði Sesseljuson[1], Raffaele Nardone[2], Eugen Trinka[3], Aljoscha Thomschewski[3], and Yvonne Höller[1]

1 Faculty of Psychology, University of Akureyri, Akureyri, Iceland

2 Department of Neurology, Hospital of Merano (SABES-ASDAA), Merano, Italy

3 Department of Neurology, Neurocritical Care and Neurorehabilitation, Member of European Reference Network EpiCARE, Center for Cognitive Neuroscience, Christian Doppler University Hospital, Paracelsus Medical University, Salzburg, Austria

Correspondence: vugar@unak.is*

USA

Modality	Links and Sources	Notes
EEG	1.https://journals.lww.com/clinicalneurophys/fulltext/2016/08000/american_clinical_neurophysiology_society.3.aspx **(scientific article; American Clinical Neurophysiology Society guidelines)**<br><br>2.https://abret.org/application/files/5517/2321/4314/REEGT_Handbook_2024_ADA.pdf **(neurodiagnostic credentialing handbook)**<br><br>3.https://www.childrenshospital.org/treatments/electroencephalograms#:~:text=Small%2C%20non%2Dinvasive%20electrodes%20(,and%20may%20become%20%22registered.%E2%80%9D **(hospital website)**	Such an individual is often referred to as a neurophysiologist or electroencephalographer. Psychiatrists, neurosurgeons, and psychologists may also interpret EEGs but to do so, like neurologists, they require special EEG training. Board certification is available in EEG and other aspects of neurophysiology from several organizations. Similarly EEG technologists should have special training in EEG and may become "registered."
Brain MRI	https://gravitas.acr.org/PPTS/GetDocumentView?docId=73 **(American College of Radiology guidelines)**	Interpreting brain MRI results is a specialized task typically performed by a radiologist, a doctor trained in reading and interpreting medical images. Brain MRI scans are read by subspecialized neuroradiologists that have undergone additional training in interpreting brain scans during a fellowship.
TCD	https://onlinelibrary.wiley.com/doi/10.1002/jum.16234 **(scientific article; The American Institute of Ultrasound in Medicine)**	Physicians interpreting or performing this type of ultrasound examination should meet the specified AIUM Training Guidelines[25] in accordance with AIUM accreditation policies.[26]<br>Sonographers performing the ultrasound examination should be appropriately credentialed[27] in the specialty area in accordance with AIUM accreditation policies.[26]<br>Physicians not personally performing the examination must provide supervision, as defined by the Centers for Medicare and Medicaid Services Code of Federal Regulations 42 CFR §410.32.[28]
TMS	https://www.cms.gov/medicare-coverage-database/view/lcd.aspx?lcdid=36469&ver=24&utm_source **(Centers for medicare and medicaid services website)**	The order for treatment (or retreatment) is written by a psychiatrist (MD or DO) who has examined the patient and reviewed the record. The psychiatrist will have experience in administering TMS therapy. The treatment shall be given under direct supervision of this psychiatrist (physician present in the area and immediately available but does not necessarily personally provide the treatment).

CANADA

Modality	Links and Sources	Notes
EEG	https://www.cnsf.org/media/4dpk34m1/update-on-minimal-standards-for-electroencephalography-in-canada-a-review-by-the-canadian-society-of-clinical-neurophysiologists.pdf<br>**(Canadian Society of Clinical Neurophysiologists guidelines)**	EEG A registered technologist with the Canadian Board of Registration of Electroencephalograph Technologists, Inc. will perform the EEG or EP test. /A neurologist (someone who specializes in nervous system disorders) interprets the recordings taken from the EEG or EP and then sends the results to your doctor within one to two weeks
Brain MRI	1. https://car.ca/wp-content/uploads/2021/12/RR-Position-Statement_forPDF-1.pdf?utm<br>**(Canadian Assoc. Of Radiologists guidelines)**<br><br>2.https://monctonmri.com/index.php/magnetic-resonance-imaging/#:~:text=Our%20Staff,the%20next%20steps%20with%20you<br>**(hospital website)**	**MRI Technologists** will perform your MRI. Following the scan, our **NB board-certified radiologists** will analyze your MRI images and prepare a detailed report.
TCD	1. https://westernsono.ca/wp-content/uploads/2020/09/TCD-Vincent-lau.pdf<br>**(scientific article)**<br><br>2.<br>http://pmc.ncbi.nlm.nih.gov/articles/PMC8052112/#:~:text=Reporting%20the%20number%20of%20standard,measured%20flow%20velocities%20is%20encouraged.&text=The%20pediatric%20intensivist%2C%20neurosurgeon%2C%20and,on%20the%20patient's%20underlying%20pathophysiology.&text=Abbreviations:%20ACA%2C%20anterior%20cerebral%20artery,referenced%20in%20the%20recommended%20protocol.&text=All%20panel%20members%20supported%20the%20finalized%20consensus%20statements%20and%20protocols.<br>**(scientific article)**	The pediatric intensivist, neurosurgeon, and/or neurologist should be involved in the process of interpretation based on the patient's underlying pathophysiology.<br>performs/ sonographers

BRAZIL

Modality	Links and Sources	Notes
EEG	1.https://pubmed.ncbi.nlm.nih.gov/34755770/#:~:text=Conclusion:%20Despite%20the%20well%2Destablished,whether%20EEG%20benchmarks%20require%20modifications<br>**(scientific article)**<br><br>2.https://abneuro.org.br/2023/03/07/curriculo-minimo-baseado-em-competencias-em-eletroencefalografia-e-epileptologia/?utm<br>**(Brazilian Neurology Academy website)**	EEG interpreted by neurologists, performed by technicians/neurologists
Brain MRI	https://telemedicinamorsch.com.br/blog/interpretacao-de-imagens-de-ressonancia-magnetica#:~:text=Quem%20pode%20fazer%20a%20interpreta%C3%A7%C3%A3o,Federal%20de%20Medicina%20(CFM).<br>**(hospital website)**	MRI performs technician interprets radiologist
TCD	https://www.proibras.com.br/doppler-transcraniano-portatil#:~:text=CLASSIFICA%C3%87%C3%83O%20DA%20ALTA%20TECNOLOGIA%20DO%20DOPPLER%20TRANSCRANIANO,neurologistas%2C%20neurocirurgi%C3%B5es%2C%20cirurgi%C3%B5es%20vasculares%2C%20radiologistas%20e%20ultrassonografistas.<br>**(hospital devices manufacturer website)**	The portable transcranial Doppler is used by anesthesiologists, intensive care physicians, neurologists, neurosurgeons, vascular surgeons, radiologists, and sonographers

ARGENTINA

Modality	Links and Sources	Notes
EEG	https://www.topdoctors.com.ar/diccionario-medico/electroencefalograma/ **(medical platform for appointments website)**	performs/ EEG technician interprets Neurologist
Brain MRI		
TCD		

UK

Modality	Links and Sources	Notes
EEG	1. https://www.kingstonandrichmond.nhs.uk/patients-and-families/patient-leaflets/electroencephalogram-eeg#:~:text=The%20EEG%20test%20is%20not,easily%20removed%20later%20with%20water. **(NHS website)** 2. https://www.epilepsy.org.uk/info/diagnosis/eeg-electroencephalogram#:~:text=The%20electrical%20signals%20look%20like,think%20you%20might%20have%20epilepsy **(British epilepsy assoc. website)** 3.https://www.bscn.org.uk/data/files/Guidelines/ANS%20BSCN%20%20Neurophys%20Guidelines%20V14%20final.pdf **(British society for clinical neurophysiology guidelines)**	Performs technician. Interprets physician .
Brain MRI	https://practiceplusgroup.com/knowledge-hub/mri-waiting-times/#:~:text=Who%20performs%20your%20MRI%20scan,trained%20in%20interpreting%20diagnostic%20images. **(health insurance company website)** https://assets.publishing.service.gov.uk/government/uploads/system/uploads/attachment_data/file/958486/MRI_guidance_2021-4-03c.pdf **(Medicine and healthcare products regulatory agency guidelines)**	Performs technician. Interprets physician.
TCD	https://www.evelinalondon.nhs.uk/resources/patient-information/transcranial-doppler-scanning.pdf **(NHS website)**	Performs technician or physician. Interprets physician.
TMS	https://www.elft.nhs.uk/transcranial-magnetic-stimulation **(NHS website)**	

GERMANY

Modality	Links and Sources	Notes
EEG	1.https://dvta.de/mtf#:~:text=Medizinische%20Technolog/%2Dinnen%20f%C3%BCr%20Funktionsdiagnostik%20(MTF)%20f%C3%BChren%20eigenverantwortlich%20funktionsdiagnostische,und%20Verantwortungsbewusstsein%20unbedingt%20erforderlich%20sind. **(Assoc. Of Technologists of Germany, website)**<br><br>2. https://www.helios-gesundheit.de/magazin/news/02/eeg-neurologie/#:~:text=Wie%20l%C3%A4uft%20eine%20EEG%2DUntersuchung,innen%20und%20die%20technische%20Assistenz. (**hospital website)**	Technician performs<br>Physician interprets
Brain MRI	1.https://www.praktischarzt.de/untersuchungen/mrt/kopf/#:~:text=MRT%20Kopf%20Befund,markiert%20ein%20H%C3%A4matom%20(Blutgerinsel). **(physician job search website)**<br>2. https://www.uniklinik-duesseldorf.de/ausbildung-karriere/ausbildungen/fachbereich-mta/mta-r-oder-mta-f-das-berufsbild/mta-r#:~:text=Medizinische/r%20Technologin/Technologe%20f%C3%BCr%20Radiologie%20(MT%2DR,die%20modernsten%20Ger%C3%A4te%20zum%20Einsatz. **(hospital website)**	Technician performs<br>Physician interprets
TCD	https://schlaganfallbegleitung.de/wissen/dopplersonographie-duplexsonographie#:~:text=Die%20Doppler%2D%20und%20Duplexsonographie%20werden,Gehirn%20mit%20Blut%20versorgen%2C%20durchgef%C3%BChrt. **(medical forum about stroke, correspondence with Deutsche Gesellschaft für Neurologie)**	Performs and interprets physician with expertise in sonogrpahy
TMS	https://www.mdpi.com/2035-8377/14/4/82<br>https://www.krankenhaus-kempen.de/neurozentrum/zentrum-fuer-neuromodulation	

AUSTRIA

Modality	Links and Sources	Notes
EEG	https://www.gesundheit.gv.at/labor/untersuchungen/funktionsdiagnostik/eeg.html#:~:text=eine%20EEG%20durchgef%C3%BChrt?-,Wo%20wird%20eine%20EEG%20durchgef%C3%BChrt?,mit%20neurologischer%20Abteilung%20durchgef%C3%BChrt%20werden. **(ministry of health website)**	Technician performs<br>Physician interprets
Brain MRI	https://www.gesundheit.gv.at/gesundheitsleistungen/berufe/gesundheitsberufe-a-z/diagnose-therapie-beratung/radiologietechnologe.html **(ministry of health website)**	Technician Performs<br>Physician interprets
TCD	https://www.gesundheit.gv.at/labor/untersuchungen/ultraschall.html **(ministry of health website)**	Physician both performs and interprets
	**Correspondence with gesundheitsportal@goeg.at ministry of health**<br>1.https://www.gesundheit.gv.at/labor/untersuchungen/ultraschall.html<br>2.https://www.gesundheit.gv.at/labor/untersuchungen/funktionsdiagnostik/eeg.html<br>3.https://www.gesundheit.gv.at/labor/untersuchungen/mrt-ct-roentgen/mrt.html	

FRANCE

Modality	Links	Notes
EEG	1. https://www.elsan.care/fr/pathologie-et-traitement/maladies-neurologiques/electroencephalogramme-EEG-deroulement-resultat#:~:text=Un%20%C3%A9lectroenc%C3%A9phalogramme%20(EEG)%20est%20un,un%20m%C3%A9decin%20sp%C3%A9cialis%C3%A9%20en%20neurologie **(website for medical appointments)**<br>2. https://www.chu-bordeaux.fr/Les-unit%C3%A9s-m%C3%A9dicales/Exploration-du-syst%C3%A8me-nerveux/EXAMENS-EEG/#:~:text=L'%C3%A9lectroenc%C3%A9phalogramme%20est%20un%20examen,par%20un%20m%C3%A9decin%20neurologue%20sp%C3%A9cialis%C3%A9. **(hospital website)**	Performs technician<br>Interprets physician

Brain MRI	https://lemedecin.fr/medical/actes/acqj002-remnographie-irm-du-crane-et-de-son-contenu-avec-injection-intraveineuse.html?utm<br>**(telemedicine platform)**	performs technician<br>interprets radiologist
TCD	https://www.ffn-neurologie.fr/explorations_neurologiques/lechographie-doppler-des-arteres-cervicales-et-transcranien/#:~:text=Qui%20le%20r%C3%A9alise%20?,cardiologie%2C%20et%20parfois%20de%20neurologie.<br>**(French federation of neurologists website)**	This examination is performed by a physician, most often a radiologist, a vascular physician, a cardiologist, or a neurologist. More recently, it can also be performed by a medical electroradiology technician. It is performed in hospitals in care departments, for example, in a neurovascular or cardiology unit, as well as in radiology departments. In the community, it is performed in radiology, vascular medicine, cardiology, and sometimes neurology offices

NETHERLANDS

Modality	Links and Sources	Notes
EEG	1. https://www.hersenstichting.nl/diagnose/eeg/#:~:text=Uitslag-,Wat%20is%20EEG?,een%20kinderarts%20het%20onderzoek%20aanvragen. **(non-profit health organization website)**<br>2. https://info.mumc.nl/pub-598#:~:text=De%20uitslag%20van%20het%20EEG,poli%20van%20uw%20behandelend%20arts.<br>(**hospital website)**	performs technician<br>interprets physician
Brain MRI	https://www.lumc.nl/afdelingen/radiologie/diagnoses-en-behandelingen/mri-scan/<br>**(hospital website)**	Performs technician<br>Interprets physician
TCD	https://www.tergooi.nl/patienteninformatie/tcd/#:~:text=Beoordeling%20en%20uitslag,bespreekt%20de%20bevindingen%20met%20u.<br>**(hospital website)**	Physician both performs and interprets
TMS	https://www.neurocaregroup.com/news-insights/in-which-countries-is-tms-therapy-insured<br>https://www.amsterdamumc.nl/nl/patienteninformatie/repetitieve-transcraniele-magnetische-stimulatie-rtms.htm	insured

DENMARK

Modality	Links and Sources	Notes
EEG	1. https://www.molholm.dk/specialer/neurofysiologi/info/eeg-undersogelse-voksen<br>2.https://www.molholm.dk/specialer/neurofysiologi/eeg/#:~:text=Efter%20unders%C3%B8gelsen%20kan%20du%20vaske,videodokumentation.<br>**(hospital website; correspondence with minitstry of health sst@sst.dk)**	Performs technician<br>Interprets physician
Brain MRI	1.https://phdanmark.dk/mr-scanning-paa-privathospital/<br>2. https://www.helkropsmr.dk/hvad-tilbyder-vi/?gad_source=1&gad_campaignid=21518186352&gbraid=0AAAAACKKUI8SfoKqtofoXF_ZPQVMpCWEV&gclid=Cj0KCQjwzt_FBhCEARIsAJGFWVlm3pv-5PlERkolZtfnA-XHV0AH9LtyRzYSAL3Cf0Qcj7_isnAwdLsaAlx_EALw_wcB<br>**(hospital website; correspondence with minitstry of health sst@sst.dk)**	Performs technician<br>Interprets physician
TCD	**(correspondence with minitstry of health sst@sst.dk)**	Performs technician<br>Interprets physician
TMS	https://tmsklinik.dk/<br>https://www.tandfonline.com/doi/full/10.1080/08039488.2024.2419620	

ITALY

Modality	Links and Sources	Notes
EEG	https://istitutosantachiara.it/elettroencefalogramma-cose-e-a-cosa-serve/<br>**(hospital website)**	Performs technician.<br>Interprets physician
Brain MRI	1.https://www.alliancemedical.it/rm<br>**(healthcare services company website)**<br>2. https://www.my-personaltrainer.it/salute/risonanza-magnetica-encefalo.html#:~:text=Come%20ogni%20tipo%20di%20risonanza,spetta%20a%20un%20medico%20radiologo.<br>**(health forum)**	performs technician<br>interprets physician
TCD	https://www.centralino.it/sanitario/enciclopedia-medica/Prestazione/6698604ff346c4608ef2685a/ECO-COLOR-DOPPLER-TRANSCRANICO<br>**(helthcare services website)**	**Neurologists** : for the diagnosis and monitoring of neurological diseases related to blood circulation.<br>**Neurosurgeons** : During and after surgery to monitor cerebral blood flow.<br>**Emergency Room Physicians** : for a rapid initial assessment in cases of suspected stroke.<br>**Radiology and Ultrasound Technicians** : specifically trained to perform this procedure and interpret the results

SPAIN

Modality	Linksn and Sources	Notes
EEG	**Correspondence with Spain Neurology Society**	Physician both performs and interprets
Brain MRI	1.https://www.cun.es/enfermedades-tratamientos/pruebas-diagnosticas/resonancia-magnetica<br>**(hospital website)**	Performs technician<br>Interprets physician
TCD	**Correspondence with Spain Neurology Society**	Physician both performs and interprets

POLAND

Modality	Links	Notes
EEG	1.https://www.doz.pl/czytelnia/a15135-EEG__na_czym_polega_elektroencefalografia_Jakie_sa_wskazania_i_jak_przebieg a_badanie_EEG#:~:text=pacjenta%20na%20kamerze.-,EEG%20%E2%80%93%20interpretacja%20wynik%C3%B3w,maj%C4%85%20one%20w%C3%B3wczas%20inny%20przebieg).//https://www.neuro-care.pl/baza-wiedzy-i-opinie-pacjentow/diagnostyka#:~:text=Jak%20interpretowa%C4%87%20badanie%20EEG?,zdrowych%2C%20bez%20%C5%BCadnych%20objaw%C3%B3w%20neurologicznych.<br>**(pharmacy website)**<br>2. https://www.mp.pl/pacjent/badania_zabiegi/314780,badanie-eeg#:~:text=Badanie%20EEG%20%E2%80%93%20wyniki,d%C5%82u%C5%BCej%20lub%20inne%20badanie%20m%C3%B3zgu.<br>**(health forum)**	Performs technician<br>Interprets physician
Brain MRI	1.https://inforadiologia.pl/informacje,kto-nas-bada-w-pracowniach-radiologicznych--,62.html#:~:text=Obs%C5%82ug%C4%85%20aparatury%20radiologicznej%20zajmuj%C4%85%20si%C4%99,posiadaj%C4%85%20zawodowy%20tytu%C5%82%20technik%20elektroradiologii.<br>**(health forum)**<br>2. https://zdrowie.pzu.pl/poradnik-o-zdrowiu/szczegoly/radiolog-lekarz-od-badan-obrazowych-czym-sie-zajmuje#:~:text=Radiolog%20to%20lekarz%2C%20kt%C3%B3ry%20zajmuje,zmiany%2C%20dokonuje%20r%C3%B3wnie%C5%BC%20ich%20pomiar%C3%B3w.<br>**(health forum)**	Performs technician<br>Interprets physician
TCD	https://www.arkmedic.pl/usg-doppler-przezczaszkowe-tccd/<br>**(hospital website)**	Interprets physician

SERBIA

Modality	Links and Sources	Notes
EEG	1. https://www.apollohospitals.com/sr/diagnostics-investigations/eeg-test<br><br>2. https://www.apollohospitals.com/sr/diagnostics-investigations/electroencephalogram<br>**(hospital websites)**	performs technician<br><br>interprets physician
Brain MRI		
TCD		

TURKEY

Modality	Links and Sources	Notes
EEG	1.https://www.medicalpark.com.tr/eeg-cekimi-zararlari/hg-3886<br>2. https://www.livhospital.com/eeg-nedir-neden-cekilir#:~:text=EEG%20%C3%A7ekimi%2C%20genellikle%20hasta%20rahat,y%C3%B6netim%20planlar%C4%B1n%C4%B1n%20belirlenmesine%20yard%C4%B1mc%C4%B1%20olur.<br>**(hospital websites)**	performs technician<br><br>interprets neurologist
Brain MRI	1. https://www.centermed.com.tr/tr/manyetik-rezonans-mri-uc-tesla<br>2. https://sertifika.subu.edu.tr/radyoloji-teknisyeniteknikeri-nedir-ne-is-yapar-maasi-nedir<br>**(hospital websites)**	performs technician<br>interprets physician
TCD	https://www.drrengin.com/tr/transfontanel-ultrason#:~:text=Transfontanel%20Ultrasonu%20Kim%20Yapmal%C4%B1d%C4%B1r?,iyi%20tedaviyi%20veya%20%C3%B6nlemi%20alacakt%C4%B1r.<br>**(hospital website)**	Performs and interprests physician

RUSSIA

Modality	Links and sources	Notes
EEG	**Correspondence with Russian Neurology Society; national law**	Performs „functional diagnostics nurse" (technician)<br><br>Interprets physician
Brain MRI	**Correspondence with Russian Neurology Society; national law**	Performs technician<br>Interprets Physician
TCD	**Correspondence with Russian Neurology Society; national law**	Physician both performs and interprets

INDIA 2

Modality	Links and sources	Notes
EEG	https://www.medicoverhospitals.in/hi/articles/eeg-test-cost-in-india<br>**(hospital website)**	Physician both performs and interprets
Brain MRI	https://www.tenderpalm.com/brain-mri-in-lucknow-india#:~:text=Who%20performs%20a%20brain%20MRI,performance%20of%20an%20MRI%20scan<br>**(hospital website)**	Performs technician<br>Interprets physician
TCD	https://www.tenderpalm.com/transcranial-doppler-in-lucknow-india#:~:text=Generally%2C%20no%20special%20preparation%20is,in%20a%20transcranial%20Doppler%20ultrasound:<br>**(hospital website)**	Performs technician<br>Interprets physician

CHINA

Modality	Links and sources	Notes
EEG	https://pubmed.ncbi.nlm.nih.gov/34510417/v<br>**(scientific article)**	Performs technician<br>Interprets physician
Brain MRI		
TCD		

JAPAN

Modality	Links	Notes
EEG	1.https://www.musako-pediatrics.com/eeg/#:~:text=%E6%A4%9C%E6%9F%BB%EF%BC%89%E3%81%A8%E3%81%AF-,%E8%84%B3%E6%B3%A2%E6%A4%9C%E6%9F%BB%E3%81%A8%E3%81%AF,%E4%BC%9D%E9%81%94%E3%82%92%E3%81%97%E3%81%A6%E3%81%84%E3%81%BE%E3%81%99%E3%80%82 **(hospital website)** 2.https://pmc.ncbi.nlm.nih.gov/articles/PMC9515718/#:~:text=2.1.&text=All%20EEGs%20were%20recorded%20in,were%20selected%20by%20unipolar%20montage.&text=We%20considered%20that%20a%203,T6%2C%20and%20T6%E2%80%93O2. **(scientific article)**	Performs technician<br><br>Interprest physician
Brain MRI		
TCD		
TMS	Correspondence with Yoshihiro Noda https://www.sciencedirect.com/science/article/pii/S0165178124005481?via%3Dihub In Japan, clinical use of rTMS requires an order/prescription from a specialist physician. The rTMS treatments are usually carried out by technicians or nurses; however, they are performed under the supervision of a physician (meaning a doctor is always present within the facility).<br><br>By contrast, for neurophysiological experiments using single-pulse TMS, the operator does not necessarily have to be a physician. These experiments can be performed by technicians or researchers who have received training and possess the necessary knowledge and skills in TMS.	

UAE

Modality	Links	Notes
EEG	1. https://emirateshospitals.ae/services/neurology/neurology-tests-diagnosis/electroencephalography-eeg/#:~:text=The%20hospital's%20commitment%20to%20precision,provide%20specific%20instructions%20if%20required. **(hospital website)**	Performs technician<br>Interprets physician
Brain MRI	1. https://www.drhc.ae/dubai-radiology-center/mri-scan<br>2. https://braindubai.com/disease/radiology/	Performs technician<br>Interprets physician

	**(hospital website)**	
TCD		

AUSTRALIA

Modality	Links	Notes
EEG	**Correspondence with Australian and New Zealand Association of Neurologists**	Performs technician<br>Interprets Physician
Brain MRI	**Correspondence with Australian and New Zealand Association of Neurologists**	Performs technician<br>Interprets physician
TCD	**Correspondence with Australian and New Zealand Association of Neurologists**	Performs technician/physician<br>Interprets physician
TMS	https://www.ranzcp.org/clinical-guidelines-publications/clinical-guidelines-publications-library/repetitive-transcranial-magnetic-stimulation?utm_source<br>australia rTMS is prescribed by a psychiatrist with training and expertise in rTMS. The rTMS session is administered by a psychiatrist or an appropriately trained healthcare professional, such as a psychiatric nurse, under the supervision of the psychiatrist.	

ICELAND

Modality	Links	Notes
EEG		
Brain MRI	https://www.visindavefur.is/svar.php?id=5037<br>**(university of Iceland's news website)**	Performs technician<br>Interprets physician
TCD		

ALGERIA

Modality	Links	Notes
EEG		
Brain MRI		
TCD		

EGYPT

Modality	Links	Notes
EEG		
Brain MRI		
TCD		

Nigeria

Modality	Links	Notes
EEG		
Brain MRI	https://www.surjen.com/lab-test-details/mri-scan **(hospital website)**	Physician interprets
TCD		

RSA

Modality	Links	Notes
EEG	**Correspondence with Neurological Association of South Africa**	Performs technician<br>Interprets physician
Brain MRI	**Correspondence with Neurological Association of South Africa**	Performs technician<br>Interprets physician
TCD	**Correspondence with Neurological Association of South Africa**	Physician both performs and interprets
